# Whole genome sequencing of 18 economically important aphid pests with photographic vouchers for taxonomic validation

**DOI:** 10.1186/s12863-026-01406-w

**Published:** 2026-01-29

**Authors:** Virgile Ballandras, Louise McNamara, James C. Carolan, Antoine Pichon, Stephen Byrne

**Affiliations:** 1 Crop Science Department, Teagasc, Carlow, R93 XE12 Ireland; 2 Department of Biology, Maynooth University, Maynooth, Co. Kildare W23 F2H6 Ireland; 3 South East Technological University, Summerhill Road Campus, Wexford, Ireland

**Keywords:** Aphids, Genome sequence data, Metabarcoding, Metagenomics

## Abstract

**Objectives:**

Accurate molecular identification in insect monitoring programs relies on validated genomic references, yet many pest species remain underrepresented or incorrectly annotated in public databases. This Data Note provides a curated genomic resource for 18 economically important aphid pests. For each species, we generated whole-genome shotgun sequences and captured high-resolution photographic vouchers of the sequenced individuals to ensure taxonomic verification. Specimens were collected from field or suction trap networks to incorporate intraspecific variation. This dataset will support the development of reliable DNA barcoding, metabarcoding, and mitochondrial metagenomic assays, and contribute to improved reference libraries for aphid pest surveillance.

**Data description:**

This dataset includes whole-genome shotgun sequencing data for 18 agriculturally important aphid pest species selected from suction trap monitoring programs. Specimens were morphologically identified using standard aphid identification keys, and diagnostic traits were documented with high-resolution Leica Flexacam C3 images to provide taxonomic verification. For each species, pooled individuals (up to 15 per species) were used for DNA extraction using the Monarch^®^ Genomic DNA Purification Kit. Illumina 150 bp paired-end sequencing (10.1–22.7 Gb per species) was performed by Novogene. These data enable extraction of Cytochrome Oxidase I (COI) barcodes, mitochondrial genomes, and associated endosymbiont sequences.

## Objective

Insect identification in monitoring programs is predominantly based on morphological examination. Taxonomists rely on identification keys derived from physical characteristics of voucher specimens, typically curated in museum collections, to assign taxonomic identifiers (family, genus, species) to collected insects. In recent years, DNA metabarcoding has been proposed as a complementary approach for large-scale insect monitoring. However, the reliability of metabarcoding depends on access to accurately vouchered reference sequences. Without these, barcode data cannot be confidently linked to a given taxon.

Although public genetic databases contain many insect sequences, coverage is uneven and biased toward a few well-studied taxa. The limited sequencing of numerous species leads to an underestimation of their genetic diversity, while a small subset of frequently sequenced taxa dominates current data resources. Furthermore, taxonomic misassignments are common, often arising from inaccurate morphological identification of specimens prior to sequencing or contamination during sample processing. These errors can compromise biodiversity assessments by misrepresenting species boundaries and community composition.

This study aimed to create a curated genomic resource to support the development of DNA barcoding, metabarcoding, and metagenomic approaches for agricultural pest surveillance. We generated whole-genome shotgun sequence data for 18 key aphid pest species of economic importance. For each specimen, we captured high-quality digital photographs to provide permanent morphological vouchers for taxonomic verification. Most specimens were alate, as they were obtained from either suction traps or yellow-pan traps. To represent intraspecific diversity, aphids were sampled from suction traps used in insect monitoring networks, covering multiple geographic locations and time points. This dataset will enhance the accuracy of species assignments in molecular diagnostics, support assay design targeting COI or mitochondrial genomes, and contribute to robust reference libraries for aphid pest identification in crop protection programs.

## Data description

In this study, we generated whole-genome sequencing data for 18 aphid species of agricultural importance. Species were selected based on their relevance to Rothamsted’s Insect Survey and those commonly detected in Irish suction trap monitoring networks. The species included were *Acyrthosiphon pisum*, *Aphis fabae*, *Aphis nasturtii*, *Brachycaudus helichrysi*, *Brevicoryne brassicae*, *Cavariella aegopodii*, *Cavariella theobaldi*, *Drepanosiphum platanoidis*, *Elatobium abietinum*, *Hyalopterus pruni*, *Macrosiphum euphorbiae*, *Megoura viciae*, *Metopolophium dirhodum*, *Myzus ascalonicus*, *Myzus persicae*, *Rhopalosiphum insertum (= R. oxycanthae)*, *Rhopalosiphum padi*, and *Sitobion fragariae*. These species span major crop pests affecting cereals, brassicas, legumes, and horticultural crops.

All individuals were morphologically identified using established aphid identification keys [1–4]. Key diagnostic traits for species determination (e.g. siphunculi, rostrum and cauda) were examined under a Leica M125 stereomicroscope, and diagnostic features were photographed using a Leica Flexacam C3 camera. High-resolution voucher images were recorded for every individual contributing to DNA extraction (Dataset 1 [5]), providing permanent visual documentation of taxonomic identity. Only specimens for which all required diagnostic characters were clearly visible and consistent with the identification key were retained; uncertain identifications were excluded.

Where possible, 15 individuals per species were pooled to capture intraspecific genetic diversity. In cases where fewer individuals were available from field collections, all suitable specimens were used (details provided in Data File 1 [6]), Where possible, specimens originated from suction traps or field monitoring traps, ensuring representation of natural field populations rather than laboratory colonies. The three suction traps are all located in Ireland in Counties Cork, Carlow, and Dublin.

Genomic DNA was extracted from pooled individuals per species using the Monarch^®^ Genomic DNA Purification Kit (New England Biolabs, USA). DNA quality and integrity were checked using Qubit fluorescence-based DNA measurement and gel electrophoresis prior to sequencing. Whole-genome shotgun sequencing was performed by Novogene (Cambridge, UK) using Illumina TruSeq library preparation protocols and paired-end 150 bp sequencing. For each species, between 10.1 and 22.7 Gb of raw sequence data were generated (Dataset 2 [7]), , representing sufficient coverage for downstream analysis of mitochondrial genomes, COI barcodes, and symbiont genomes Table 1.

**Table 1:** Overview of data files/data sets

Label	Name of data file/data set	File types (file extension)	Data repository and identifier (DOI or accession number)
Data file 1	Metadata associated with insect images	Text document (.txt)	Zenodo 10.5281/zenodo.17101188 [6]
Data set 1	Images of key aphid pests following identification utilising taxonomic keys	Images (.tif and .jpeg)	Zenodo 10.5281/zenodo.17100676 [5]
Data set 2	Whole Genome Shotgun sequencing of 18 aphid species	Fastq files (.fq)	European Nucleotide Archive http://identifiers.org/insdc.sra:SRP598348 [7]

This dataset provides a taxonomically verified whole-genome resource for key aphid pest species. The availability of paired voucher images and genomic data enables traceability and reproducibility, supporting accurate reference library development. The dataset can be mined to:

1. Extract COI barcodes for assay development and validation,
2. Assemble mitochondrial genomes for mitochondrial metagenomics, and.
3. Explore secondary endosymbiont diversity associated with aphid pests.

This resource will primarily serve as a foundation for improving DNA barcoding accuracy and enhancing metabarcoding and metagenomic pipelines for pest surveillance.

## Limitations

No limitations identified.

## Data Availability

The data described in this Data note can be freely and openly accessed on Zenodo under the DOI‘s [10.5281/zenodo.17100676](https://doi.org/10.5281/zenodo.17100676) [5] and [10.5281/zenodo.17101188](10.5281/zenodo.17101188) [6 ], and the ENA under accession SRP598348 [7].

## Abbreviation

COI: Cytochrome Oxidase I
